# Supplementary figures and images for: Identification of Vietnamese Flea Species and Their Associated Microorganisms Using Morphological, Molecular, and Protein Profiling
Source: Microorganisms. 2023 Mar 9;11(3):716. doi: 10.3390/microorganisms11030716 (PMC10055665; doi:10.3390/microorganisms11030716)

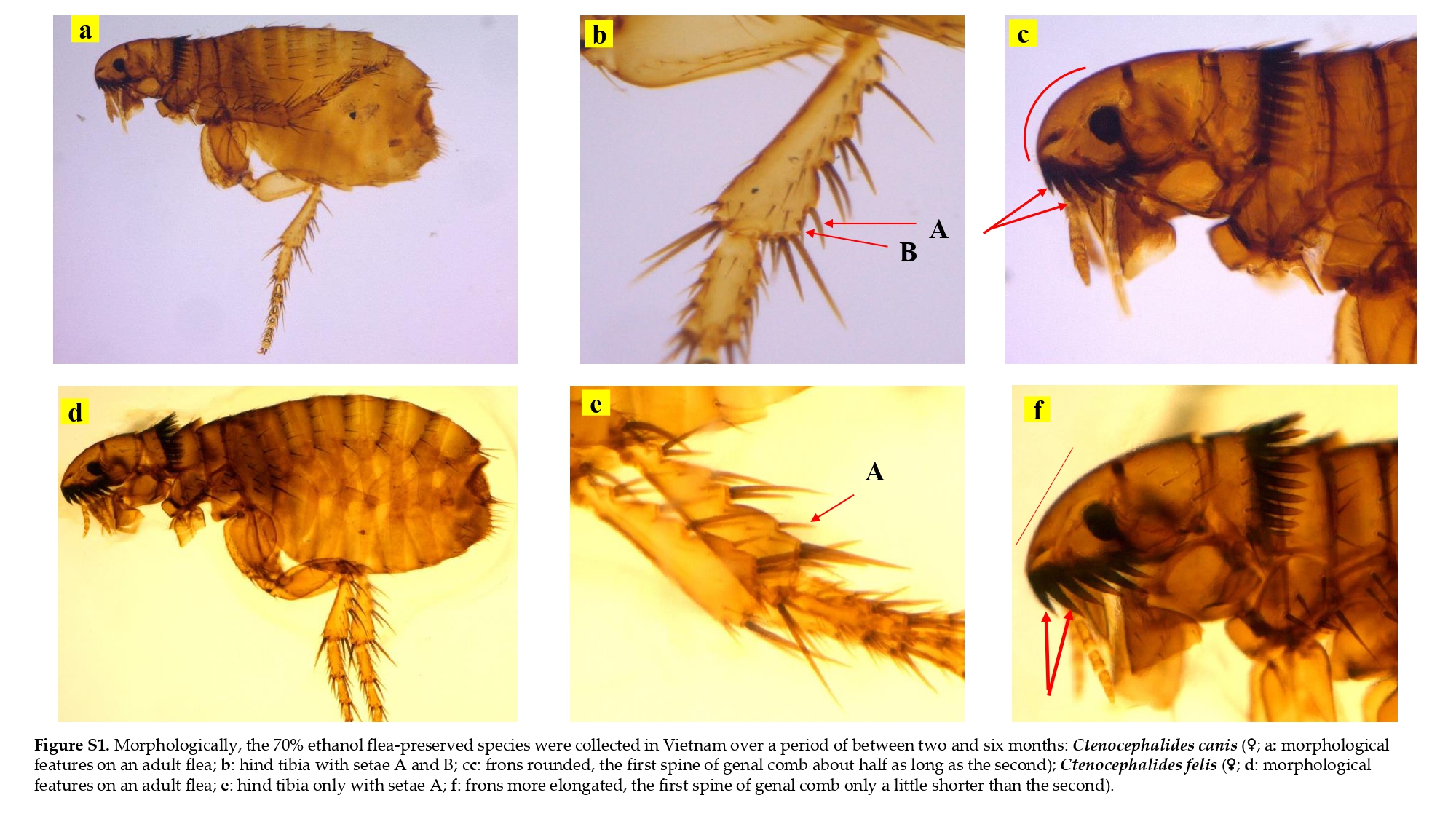

Supplement: Supplementary file 1 [file microorganisms-11-00716-s001.zip › microorganisms-2220330-supplementary/FIGURE S1_merged_page-0001.jpg]

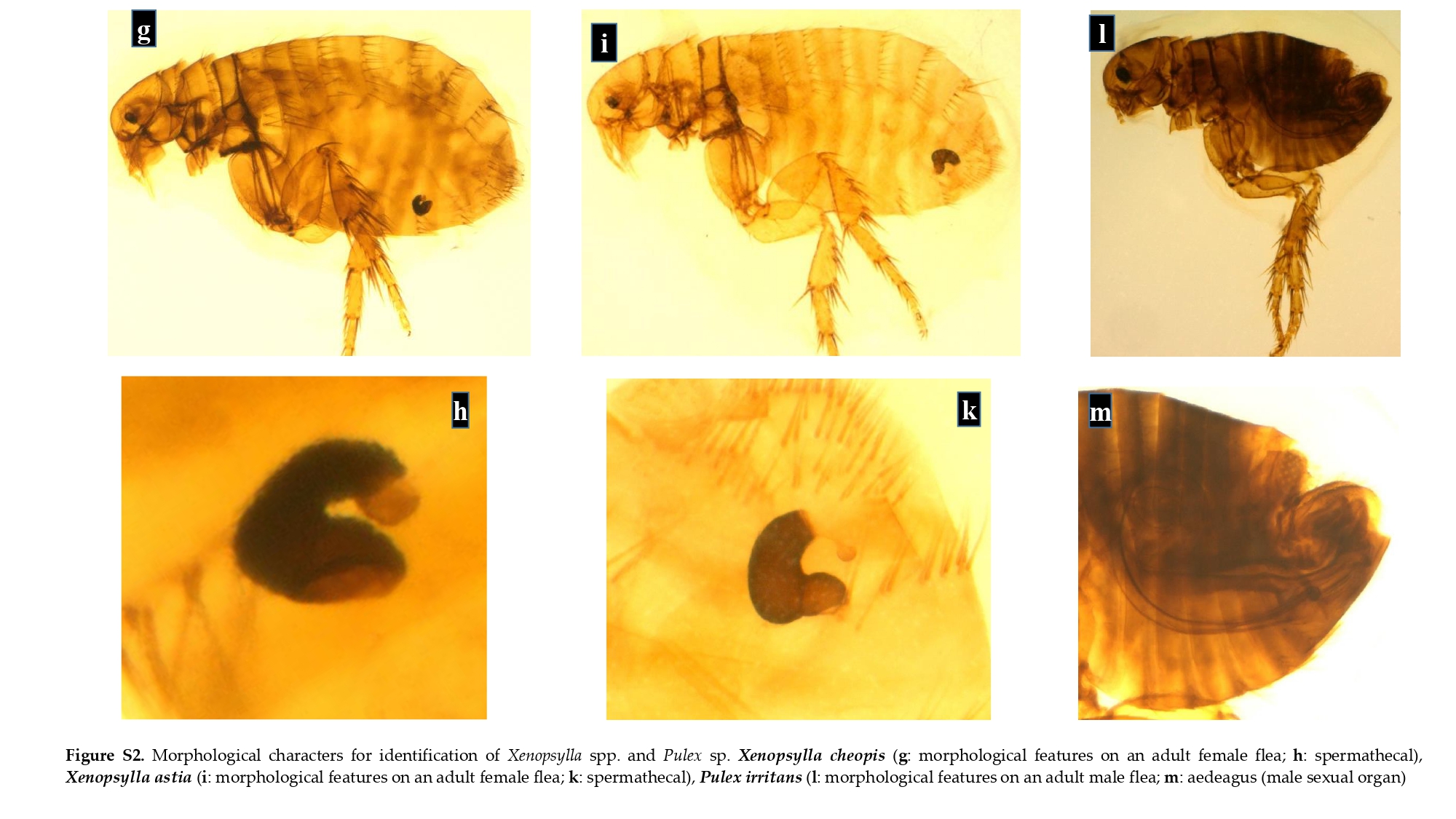

Supplement: Supplementary file 1 [file microorganisms-11-00716-s001.zip › microorganisms-2220330-supplementary/FIGURE S1_merged_page-0002.jpg]
